# Knowledge, attitudes and practices on Rift Valley fever among agro pastoral communities in Kongwa and Kilombero districts, Tanzania

**DOI:** 10.1186/s12879-015-1099-1

**Published:** 2015-08-21

**Authors:** Sasita S. Shabani, Mangi J. Ezekiel, Mohamed Mohamed, Candida S. Moshiro

**Affiliations:** Health Department, Chunya District Council, Mbeya, Tanzania; Tanzania Field Epidemiology and Laboratory Training Programme, Dar es Salaam, Tanzania; Quality Assurance Department, Ministry of Health and Social Welfare, Dar es Salaam, Tanzania; Department of Behavioural Sciences, Muhimbili University of Health and Allied Sciences, PO Box 65015, Dar es Salaam, Tanzania; Department of Epidemiology and Biostatistics, Muhimbili University of Health and Allied Sciences, Dar es Salaam, Tanzania

## Abstract

**Background:**

Rift valley fever (RVF) is a re-emerging viral vector-borne disease with rapid global socio-economic impact. A large RVF outbreak occurred in Tanzania in 2007 and affected more than half of the regions with high (47 %) case fatality rate. Little is known about RVF and its dynamics. A cross sectional study was conducted to assess the knowledge, attitudes and practices regarding RVF in Kongwa and Kilombero districts, Tanzania.

**Methods:**

We conducted a cross sectional survey among a randomly selected sample of individuals in 2011. We administered questionnaires to collect data on demographic characteristics, knowledge on symptoms, mode of transmission, prevention, attitudes and health seeking practices.

**Results:**

A total of 463 community members participated in this study. The mean (±SD) age was 39.8 ± 14.4 years and 238 (51.4 %) were female. Majority of respondents had heard of RVF. However, only 8.8 % knew that mosquitoes were transmitting vectors. Male respondents were more likely to have greater knowledge about RVF. A small proportion mentioned clinical signs and symptoms of RVF in animals while 73.7 % mentioned unhealthy practices related to handling and consumption of dead animals. Thorough boiling of milk and cooking of meat were commonly mentioned as preventive measures for RVF. Majority (74.6 %) sought care for febrile illness at health facilities. Few (24.3 %) reported the use of protective gears to handle dead/sick animal while 15.5 % were consuming dead animals.

**Conclusion:**

Our study highlights the need to address the limited knowledge about RVF and promoting appropriate and timely health seeking practices. Rift valley fever outbreaks can be effectively managed with collaborative efforts of lay and professional communities with a shared perception that it poses a serious threat to public and animal health. The fact that this study was conducted in “high risk transmission areas” warrants further inquiry in other geographic regions with relatively low risk of RVF.

## Background

Rift Valley Fever (RVF) is a viral vector borne disease caused by Rift Valley Fever Virus, a member of the genus *Phlebovirus* in the family *Bunyaviridae* that primarily affects domestic ruminants, causing large epizootics with high mortality rates in young animals and abortions [1–4]. Furthermore the RVF virus has been demonstrated to affect a wide range of wild animals including buffaloes, rhinoceros, kudu, impala, elephants and hartebeest [5]. The virus is transmitted by bite of infected mosquitoes (*Aedes* spp) and possibly by bites of other blood sucking insects such as sand fly of *phlebotomus* spp. Transmission to humans occurs through direct contact with infected animal tissues, blood or other body fluids and less commonly by mosquito bites. Furthermore, infection through aerosol transmission of RVF virus may occur as a result of contact with laboratory specimens containing the virus and unpasteurised milk from infected animals [3, 4]. However, currently there is no evidence of person to person transmission of Rift Valley Fever disease [4, 6–8]. The disease occurrences follow the unusual trend of heavy rainfall leading to flooding. The flooding provides conducive environment for dormant eggs (infected by Rift Valley fever virus) to hatch and rapidly multiply and become prominent mosquitoes populations that transmit virus to animals and subsequently from animals to humans [9–11]. In humans, RVF infection is typically asymptomatic or causes influenza like illness accompanied by fever and headache and occasionally leads to serious complications such as haemorrhagic syndromes, retinitis, encephalitis and death [6, 10, 12–15].

Since it was first detected in 1930s, multiple outbreaks have been reported in different parts of Africa and the Middle East [16–20]. Therefore, RVF is not only a threat in Africa and Middle East but holds the potential to reach other parts of the world including the western hemisphere [19]. RVF is increasingly becoming a global threat as it is being reported in other parts of the world beyond the great African Rift Valley region. In Tanzania more than four (4) RVF outbreaks have occurred in 10–20 years intervals [20]. The most recent, largest and well documented RVF outbreak occurred in 2007. The outbreak resulted in 309 human cases and 144 deaths (CFR = 47 %). It affected 52.4 % (n = 21) of Tanzania mainland regions and reports show that 72.7 % of the reported cases had concurrent infections in animals and humans [10]. Apart from human loss, the outbreak resulted in serious economic loss at individual and national level. About 135,442 ruminants (48,700 cattle, 55,276 goats and 31,466 sheep) were affected of which 34 % were aborted and 37 % died [21].

Occurrence of large outbreaks of RVF in parts of Africa and Arabian Peninsula has increased the virological and entomological knowledge regarding the RVF [18, 20, 21–23]. However, there is limited information regarding community knowledge, attitudes and practices with regard to RVF. Effective early warning and surveillance system for timely outbreak response require both livestock keepers and community to have adequate knowledge to detect the RVF disease in advance [24]. Previous evidence shows that control measures that were taken in Tanzania and Kenya mirrored the multidimensional nature of RVF. It included closing livestock markets and butcheries, imposing livestock movement controls and quarantines, and providing advice warning against drinking raw milk, slaughtering animals, or eating uninspected meat. Following principles of one health, there is a need for inter disciplinary collaboration in tackling zoonoses such as RVF. Collaboration is not only confined to outbreak control but also in strengthening outbreak preparedness [7].

Studies conducted elsewhere revealed low knowledge following RVF outbreaks. A study conducted in Sudan revealed that 82 % of livestock owners did not know modes of transmission and 70 % could not identify the correct vector for RVF [21, 24]. Other studies done among agro-pastoralist communities in Kenya and Tanzania showed limited awareness of RVF signs and symptoms in both animals and humans [23].

In light of unpredictability of RVF outbreaks and the available evidence of its public health and social economic impact, there is a need to assess knowledge, attitudes and practices in order to provide the basis for health education and promotion interventions. This article presents findings from a cross sectional survey to assess knowledge, attitudes and practices on RVF among agro-pastoral communities in rural Tanzania. Specifically this study was designed to assess knowledge regarding cause, symptoms, transmission and prevention of RVF community members, to describe attitudes towards RVF among community members, to determine perceived risk of RVF among community members and to determine health seeking practices in the event of RVF related symptoms among study population.

## Methods

### Study design and setting

We conducted a community based cross sectional study between November and December 2011 in Kongwa and Kilombero districts of Dodoma and Morogoro regions respectively. These regions represent areas that reported the highest number of RVF cases during the 2007 outbreak [10, 23]. Kongwa district is among the six districts of Dodoma region with a population of 301,566. The district is made up of 3 divisions, 23 wards, 74 villages and 286 hamlets. It is served by 41 health facilities (1 hospital, 3 health centres and 37 dispensaries). Agriculture and livestock keeping are the predominant economic activities. Kilombero district is among the five districts of Morogoro region with a population of 321,611 inhabitants. It is made up of 5 divisions, 23 wards, 76 villages and 360 hamlets. It is served by 52 health facilities (2 hospitals, 4 health centres and 46 dispensaries). Agriculture and livestock keeping are the predominant economic activities.

### Study participants

Assuming the proportion of participants knowledgeable on RVF is 50 %, absolute precision of 5 %, and 15 % non-response rate, the minimum sample size was 460 individuals. Multistage sampling technique was used to select participants from the two districts. Two divisions were selected randomly from each district. We stratified all wards in each selected division into rural and urban settings. One ward was selected randomly from each stratum, hence obtaining two wards from each division. A sampling frame of all villages from each of the 4 wards was obtained. Three villages were drawn randomly from rural and urban wards. From each selected village, we obtained a list of hamlets. Half of the available hamlets were randomly selected. A total of 39 households were selected systematically from the selected hamlets. Efforts were made to interview the head of the household, male or female aged 18 years and above; otherwise we interviewed an informed member of the household.

### Data sources and measures

A semi-structured questionnaire with both open and closed-ended questions was designed. The questionnaire was translated from English to Swahili language. Information gathered included demographic characteristics (age, sex, education, occupation, religion and marital status), knowledge about vector spreading RVF, symptoms and signs of RVF in humans and animals, and transmission modes, attitude towards RVF (perceived risk of contracting the RVF) and preventive practices against RVF (handling sick/dead animals and health seeking behaviour in febrile illness).

Prior to data collection, the Swahili version of questionnaire was pretested at a different (other then study areas) location to test the validity of questions and the translation. The questionnaire was then back translated into English and reviewed by an independent researcher (who was not part of the study team) to ensure that the questions were appropriate.

Scores ranging from zero to two were assigned to correct responses depending on the nature of the question. A score of 1 was assigned if the respondent was able to mention the vector responsible for spreading RVFV and 0 if the answer was incorrect. A score of 2 was assigned if the respondent was able to mention 3 or more symptoms of RVF in animals, a score of 1 if a respondent mentioned 1–2 symptoms and a score of zero if the answer was not provided or incorrect. A similar scoring method was used for the questions on symptoms of RVF in humans, transmission methods and preventive measures against RVF. If all answers were correct, the total score would be 9 for RVF knowledge and 4 for practice. A respondent was categorized as knowledgeable about RVF if he/she obtained a score of 5 or more out of 9 (55 % cut-off point). For practice, a respondent would be classified to have good practice if he/she obtained a score 2 or more out of 4 (50 % cut-off point).

Attitudes and perceived risks were measured using a five point Likert scale system. The response categories on each item ranged from 1 (strongly disagree) to 5 (strongly agree). For cross-tabulation analysis each item was dichotomized into (1) strongly disagree/disagree/unsure and (2) strongly agree/agree.

With regards to health seeking behaviour, participants were asked what actions they take when faced with an episode of fever. Our interest was to assess the first point of care that individuals would take as part of timely management and response to one of the major/early symptoms of RVF in humans. Therefore, we did not intend to examine factors influencing decisions to seek care at different sources/options of available health care for individuals with fever, which might be explained by different behavioural models including the Health Belief Model.

### Statistical methods

Comparison of proportions for categorical variables (socio-demographics, knowledge and practices) was done using the Chi-square test or Fisher’s exact test where appropriate. The outcome variables were knowledge about RVF and preventive practices against RVF. The relationship between various factors and knowledge about RVF or practice while taking into account potential confounding were examined using logistic regression. Odds ratios (ORs) and their corresponding 95 % confidence intervals (CIs) were estimated. All factors with *P <* 0.20 in the univariate analyses were included in the multivariate model. A two-sided P value of less than 0.05 was considered statistically significant. All statistical analyses were performed using the statistical software package SPSS version 17.

### Ethical considerations

The study protocol was approved by the Institution Review Board of Muhimbili University of Health and Allied Sciences. Permission to conduct the study was sought from Kongwa to Kilombero district authorities. Written informed consent was obtained from each individual participant before the commencement of face to face interview. In the circumstance that a participant was illiterate, verbal consent was sought.

## Results

### Participant characteristics

A total of 463 adults participated in the survey. The mean age of respondents was 40 years (range 18 to 87 years). Table 1 presents the demographic characteristics of participants in the Kongwa and Kilombero. About 51.4 % of respondents were females, one quarter were less than 30 years, 79.1 % married and 90.5 % were Christians. A slightly higher percentage of respondents in Kilombero were peasants (91.6 %) and had primary education (68.9 %). The distribution of the other characteristics was similar in the two districts.

**Table 1:** Socio-demographic characteristics of participants by district

Variable	Kongwa	Kilombero	Total
	(n = 238)	(n = 225)	(n = 463)
	No (%)	No (%)	No (%)
Sex			
Male	114 (47.9)	111 (49.3)	225 (48.6)
Female	124 (52.1)	114 (50.7)	238 (51.4)
Age group (years)			
18-29	58 (24.4)	59 (26.2)	117 (25.3)
30-39	76 (31.9)	72 (32.0)	148 (32.0)
40-49	44 (18.5)	47 (20.9)	91 (19.7)
50-59	26 (10.9)	21 (9.3)	47 (10.2)
60+	34 (14.3)	26 (11.6)	60 (13.0)
Education level			
None	94 (39.5)	56 (24.9)	150 (32.4)
Primary	131 (55.0)	155 (68.9)	286 (61.8)
Secondary+	13 (5.5)	14 (6.2)	27 (5.8)
Marital status			
Single	20 (8.4)	23 (10.2)	43 (9.3)
Married	191 (80.3)	175 (77.7)	366 (79.1)
Widow/divorced	27 (11.3)	27 (12.0)	54 (11.7)
Occupation			
Peasants	198 (83.2)	206 (91.6)	404 (87.3)
Petty traders	11 (4.6)	5 (2.2)	16 (3.5)
Civil servants	6 (2.5)	10 (4.4)	16 (3.4)
Pastoralist	23 (5.8)	4 (1.8)	27 (5.8)
Religion			
Christian	226 (90.5)	193 (85.8)	419 (90.5)
Islam	9 (3.8)	28 (12.4)	37 (8.0)
Pagan	3 (1.2)	4 (1.8)	7 (1.5)

### Knowledge of RVF

Most of the respondents (97.6 %) had heard about RVF. The reported sources of information on RVF are shown in Table 2. The main source of information was radio (70.8 %) followed by friends (20.1 %), community meetings (14.4 %) and health/veterinary workers (8.0 %). Differences between the districts were observed with radio having a higher percentage in Kilombero (*p* = 0.01) while community meetings and health personnel were mentioned more frequently in Kongwa. Multivariate analysis revealed that male sex (*p* < 0.01), primary education (*p* = 0.01) and residing in Kilombero district (*p* = 0.03) were associated with an increased likelihood of obtaining RVF information from a radio.

**Table 2:** Sources of information on Rift valley fever reported by study respondents

Source of information	Total	Kongwa	Kilombero	P value
	(n = 452)	(n = 237)	(n = 215)	
	No (%)	No (%)	No (%)	
Radio	320 (70.8)	155 (65.4)	165 (76.7)	0.01
Friends	91 (20.1)	44 (18.6)	47 (21.9)	0.38
Community meetings	65 (14.4)	51 (21.6)	14 (6.5)	<0.001
Veterinary/health personnel	36 (8.0)	31 (13.1)	5 (2.3)	<0.001
Newspaper	19 (4.2)	10 (4.2)	9 (4.2)	1.0
Television	15 (3.3)	6 (2.5)	9 (4.2)	0.31
Health campaign	13 (2.9)	11 (4.6)	2 (0.9)	0.02

Responses to knowledge questions are summarized in Table 3. Few respondents (8.8 %) correctly knew mosquitoes as a vector of RVF, 10.1 % in Kongwa and 7.4 % from Kilombero. Moreover, a small proportion could mention the clinical signs and symptoms of RVF in animals. A higher proportion of respondents in Kilombero mentioned sudden death (21.9 %) and wasting (11.0 %) as symptoms of RVF in animals. When respondents were asked about transmission methods in humans, 73.7 % mentioned consuming meat of dead animal and a significantly higher proportion in Kongwa mentioned consuming milk from sick animals (32.9 %). With regards to symptoms of RVF in humans, 15.5 % knew haemorrhage as one of the symptoms. Other symptoms were less known by the respondents. When asked about preventive practices, about one third mentioned thorough boiling of milk and cooking of meat with higher percentages reported in Kongwa compared to Kilombero. Slightly more than half of the respondents knew of the need to avoid eating dead carcasses.

**Table 3:** Proportion of respondents with knowledge about RVF transmission, symptoms and prevention

Variable	Total	Kongwa	Kilombero	P value
	N = 452	N = 237	N = 215	
Vector spreading RVF				
Mosquito	40 (8.8)	24 (10.1)	16 (7.4)	0.31
Housefly	7 (1.5)	0 (0.0)	7 (3.3)	<0.01
Tsetse fly	28 (6.2)	6 (2.5)	22 (10.2)	<0.01
Tick	4 (0.9)	4 (1.7)	0 (0.0)	0.06
Symptoms of RVF in animals				
Abortion in pregnant animals	7 (1.5)	6 (2.5)	1 (0.5)	0.09
High young animal mortality	5 (1.1)	5 (2.1)	0 (0.0)	0.03
Wasting	39 (8.6)	26 (11.0)	13 (6.0)	0.06
Sudden death	73 (16.3)	52 (21.9)	21 (9.8)	<0.0001
Diarrhoea	9 (2.0)	5 (2.1)	4 (1.9)	0.88
Transmission in humans				
Mosquito bite	6 (1.3)	3 (1.3)	3 (1.4)	0.93
From another person	12 (2.7)	8 (3.4)	4 (1.9)	0.33
Consuming meat of dead/sick animal	330 (73.7)	171 (73.4)	159 (74.0)	0.89
Consuming milk of sick animal	126 (27.9)	78 (32.9)	48 (22.3)	0.01
Touching aborted foetus	2 (0.4)	2 (0.8)	0 (0.0)	0.19
Contact with infected animal blood	18 (4.0)	11 (4.6)	7 (3.3)	0.48
Symptoms of RVF in humans				
Headache	7 (1.5)	4 (1.7)	3 (1.4)	0.80
Fever	41 (9.1)	24 (10.1)	17 (7.9)	0.42
Muscle/joint pain	3 (0.7)	2 (0.8)	1 (0.5)	0.69
Wasting	7 (1.5)	3 (1.3)	4 (1.9)	0.61
Jaundice	3 (0.7)	1 (0.4)	2 (0.9)	0.51
Haemorrhage	70 (15.5)	44 (18.6)	26 (12.1)	0.06
Preventive measures of RVF				
Thorough boiling of milk	148 (32.0)	94 (39.7)	54 (25.1)	<0.01
Thorough cooking of meat	149 (33.0)	96 (40.5)	53 (24.7)	<0.0001
Avoid eating dead carcasses	245 (54.2)	124 (52.3)	121 (56.3)	0.39
Use of protective gear	10 (2.2)	7 (1.4)	3 (3.0)	0.24
Avoid mosquito contact	3 (0.7)	2 (0.8)	1 (0.5)	0.69
Avoid contact with aborted foetus/dead animal	27 (6.0)	15 (6.3)	12 (5.6)	0.75

### Attitudes and perceived risk for RVF

Table 4 shows that majority of respondents (90.3 %) agreed that RVF was a serious disease. Ninety percent agreed that RVF posed a threat to public health as well as the local livestock economy. More than two-third (63.2 %) of respondents reported to be personally at risk of contracting RVF. Less than half (39.8 %) believed that RVF was curable. Most (83.6 %) of the respondents appreciated that the disease affects not only pastoralist but also farmers. Majority (67.5 %) reported that the disease was preventable.

**Table 4:** Attitudes and perceived risk for RVF (n = 452)

Attitude and risk statements	Number (%) agreed		
	Total (n = 452)	Kongwa (n = 237)	Kilombero (n = 215)
RVF is a serious disease	408 (90.3)	206 (86.9)	202 (94.0)
RVF is a threat to wellbeing of the community	407 (90.0)	211 (89.0)	196 (91.2)
You are at risk of getting RVF	286 (63.3)	142 (59.9)	144 (67.0)
RVF is curable	180 (39.8)	100 (42.2)	80 (37.2)
RVF affects only pastoralists	33 (7.3)	18 (7.6)	15 (7.0)
RVF transmission from animals to humans is preventable	305 (67.5)	151 (63.7)	154 (71.7)
Health facilities are prepared to handle RVF outbreaks	199 (44.0)	90 (37.9)	109 (50.7)

### Health seeking behaviour and handling practices of RVF suspect (dead) animals

Table 5 shows health seeking practices and handling of dead animals among respondents. Findings reveal that about three quarters of the respondents (74.5 %) reported that they would go to a health facilities when they have fever, with higher proportions reported in Kongwa (88.2 %) compared to 59.5 % in Kilombero (*p* < 0.001). About one quarter mentioned they would go to a drug store, with 40 % reporting from Kilombero compared to 10.5 % in Kongwa. Health belief Model has been used to explain why people choose to perform certain health related behaviours. The model has been amended to include demographic variables that may also impact people’s perceptions, self assessment of (RVF) risk assessment. Some of these variables such as educational attainment, age, marital status, sex, have been analysed in this article in relation to knowledge, attitudes and practices related to RVF. The rationale for assessing health seeking practices in this study was to specifically provide baseline information to aid in design of interventions to promote timely/early identification of key RVF symptoms and thereby contribute to improving RVF outbreak management.

**Table 5:** Health seeking and carcass handling practices among respondents (n = 452)

Variable	Total	Kongwa	Kilombero	P value
	n = 452	n = 237	n = 215	
Action taken during early stage of febrile illness				
Visit health facility	337 (74.5)	209 (88.2)	128 (59.5)	<0.001
Visit drug shop/store	110 (24.3)	25 (10.5)	85 (39.5)	<0.001
Visit traditional healer	5 (1.1)	3 (1.3)	2 (1.0)	0.77
Practice to dead animal				
Slaughter/skin	70 (15.5)	53 (22.4)	17 (7.9)	<0.001
Bury	202 (44.7)	61 (25.7)	141 (65.6)	<0.001
Inform veterinary officer	131 (29.0)	96 (40.5)	35 (16.3)	<0.001
Leave it	49 (10.8)	27 (11.4)	22 (10.2)	0.69
Practice to carcass of suspected animal				
Use protective gear	110 (24.3)	60 (25.3)	50 (23.3)	0.62
Use bear hands	180 (39.8)	95 (40.1)	85 (39.6)	0.91
Never handled	81 (17.9)	48 (20.3)	33 (15.3)	0.17
Sticks/piece of wood	50 (11.1)	22 (9.3)	28 (13.0)	0.24
Rope/hoe	31 (6.9)	12 (5.1)	19 (8.8)	0.04

When respondents were asked what they did with dead animals, 15.5 % mentioned that they slaughtered or skinned the dead animal, 45 % buried and 29 % informed the veterinary officers. Large differences were observed between the districts. Proportion of respondents reporting to have slaughtered/skinned a dead animal was significantly higher in Kongwa (22.4 %) compared to Kilombero (7.9 %). When asked on what they use for handling dead animals, about one quarter claimed use of protective gears and 40 % used bear hands.

### Factors influencing RVF knowledge and practice

Out of 452 respondents who had ever heard of RVF, 11.3 % were recorded as knowledgeable about RVF. Univariate and multivariate logistic regression results are presented in Table 6. Results showed that after adjusting for other factors, RVF knowledge was greater among male respondents and in Kongwa district. Age, education, marital status, occupation and religion were not associated with knowledge about RVF.

**Table 6:** Factors associated with knowledge about RVF transmission, symptoms and prevention (n = 452)

Variable	No. with knowledge/Total (%)	Crude OR (95 % CI)	p-value	Adjusted OR (95 % CI)	p-value
District					
Kilombero	15/215 (7.0)	Reference		Reference	
Kongwa	36/237 (15.2)	2.39 (1.27 – 4.50)	0.01	2.45 (1.29 -4.63)	0.01
Sex					
Male	32/223 (14.4)	Reference		Reference	
Female	19/229 (8.3)	0.54 (30 – 0.98)	0.04	0.54 (0.29 - 0.99)	0.047
Age group (years)					
18-29	9/111 (8.1)	Reference	0.49		
30-39	17/145 (11.7)	1.51 (0.64 – 3.52)			
40-49	14/90 (15.6)	2.09 (0.86 – 5.08)			
50-59	6/46 (13.0)	1.70 (0.57 – 5.09)			
60+	5/60 (8.3)	1.03 (0.33 – 3.23)			
Education level					
None	18/147 (12.2)	Reference	0.69		
Primary	29/279 (10.4)	0.83 (0.44 – 1.55)			
Secondary+	4/26 (15.4)	1.30 (0.40 – 4.21)			
Marital status					
Single	4/45 (8.9)	Reference	0.73		
Married	42/354 (11.9)	1.38 (0.47 – 4.05)			
Widow/divorced	5/53 (9.4)	1.07 (0.27 – 4.24)			
Occupation					
Peasant/Pastoralist	50/420 (11.9)	Reference	0.16	Reference	0.19
Business/employed	1/32 (3.1)	0.24 (0.03 – 1.79)		0.26 (0.03 – 1.93)	
Religion					
Muslim	3/37 (8.1)	Reference	0.37		
Christian	46/408 (11.3)	1.44 (0.43 – 4.88)			
Pagan	2/7 (28.6)	4.53 (0.60 – 34.2)			

Residents residing in Kongwa district had a greater likelihood of having good practices for RVF (*p* = 0.02). There were no significant associations between the other variables and practices for RVF (data not shown).

## Discussion

In this study, findings showed that overall knowledge regarding RVF vector, modes of transmission, symptoms and prevention among respondents was low (11.3 %).This result confirms findings in the study conducted in Sudan following RVF outbreak [24].Most of respondents were not aware about vectors spreading the Rift Valley Fever (RVF), signs and symptoms in animal and humans. A small proportion of respondents in both districts were able to correctly mention the principal vector. This finding is similar to a post outbreak survey conducted among pastoralist communities which showed limited and different level of knowledge among participants particularly on RVF transmission and symptoms [23]. Storm abortions in adult animals and high mortality of young animals have previously been reported to be alarming signs of RVF outbreak [4]. However, in the current study these were least reported in both study districts compared to sudden deaths of animals which was mostly mentioned in Kongwa. These results have important public health implications since late identification of these signs may affect timely response to outbreaks and foster transmission among animals and later in humans.

Our study showed that, 73.7 % knew that consuming carcasses of dead (uninspected) animals may pose a risk for RVF transmission in humans. Consumption of unsafe milk from sick animals was among the risk practices mentioned in both districts. Most of respondents were able to recall these practices because during the previous outbreak the government spread messages advising against consumption of infected/suspected animal products and also imposed a ban on trading of meat and other animal products. Other routes of transmission of RVF such as obstetric procedures, contact infected animal fluids were not familiar among study participants in both study sites. Limited awareness about other modes of transmission among study participants poses a potential risk of contracting the disease in epizootic period.

Our study showed small proportion of respondents was aware about RVF symptoms in humans. While haemorrhagic syndrome was reported by 15.5 % of respondents, few associated RVF with other febrile symptoms such as headache, fever and muscle pain. Previous studies have shown that RVF infection in humans is usually subclinical and in some cases complications arise [10, 12]. Poor knowledge on the spectrum of symptoms of RVF in human might be confused with other febrile illnesses such as malaria which is endemic in many parts of Tanzania and also in the study districts. This may have implications on timing for seeking medical attention until complications arise.

As indicated by the respondents, radio was the main reported source of information on RVF, followed by friends, community meetings and veterinary personnel. These results highlight the need to consider the role of radio as a tool for delivering RVF related information to the public.

In this study respondents had positive attitudes towards RVF. A majority (90 %) felt the disease was a serious threat to public and animal health. Majority (67 %) felt at risk of RVF infection and thought that an outbreak of the disease could affect both pastoralists and agro pastoral communities due to the close interaction between these two occupational groups. The high perceived risk to RVF may be because of the mortality and morbidities witnessed by the respondents during the previous outbreak.

In this survey, risk practice such as consuming dead/suspected animals was evident in a good number (16 %) of study respondents. Other studies have demonstrated similar findings [6, 10, 23] where individuals had reputation of eating dead/sick animals. This behaviour is an important risk factor for RVF transmission in humans. High proportion of this behaviour was reported in Kongwa (22.4 %) compared to (7.9 %) Kilombero. This may be because of cultural differences and level of awareness about healthy eating habits and also possible due to the fact that a large proportion of respondents in Kongwa were less literate compared to the counterpart district.

Early health seeking behaviour during febrile illness among respondents in this study was high. Majority (74.5 %) reported to have sought biomedical help or alternative health care (25.5 %) in case of fever. The proportion of individuals reporting to seek biomedical help for fevers are in contrast to previous studies which revealed that majority (86 %) used home remedies during febrile illnesses and sought biomedical treatment thereafter [25, 26]. This may be because members of community have huge familiarity with malaria symptoms and therefore tend to equate all episodes of fever to malaria. Furthermore, since malaria treatment costs have been subsidised by government and other partners, this might explain the motivation to visit health facilities in an event of fever as revealed in this study.

Our findings should be interpreted in light of limitations of using questionnaire based survey. However, the questionnaire was pre tested prior to actual data collection to improve the accuracy and quality of data. The study was conducted only in districts that experienced RVF outbreak and was carried out four years after the outbreak therefore the responses to some questions might be affected by recall bias. Furthermore, this study was done in outbreak prone areas thus findings may not be generalized to districts which didn’t experience the outbreak. Lastly, KAP study was appropriate to collect baseline information to inform a subsequent intervention. The KAP study was one among other approaches applied to generate quantitative data to inform the intervention. Findings from other methods used will be presented elsewhere.

## Conclusion

Control of RVF outbreaks requires timely detection informed by a good understanding of its signs and symptoms among both animals and humans. Our study highlights the need to address the limited knowledge about RVF and promoting appropriate and timely health seeking practices. Rift valley fever, being a trans-boundary zoonotic disease, is a public health problem that might be effectively managed with collaborative efforts of lay and professional communities with a shared perception that it poses a serious threat to public and animal health. Alternative health seeking behaviour and risk practice of consuming meat from dead and uninspected animals reported in this study calls for public health interventions to raise awareness and address risk practices that might precipitate RVF transmission during outbreaks in future. The fact that this study was conducted in “high risk transmission areas” warrants further inquiry in other geographic regions with relatively low risk of RVF transmission.
